# Generic affiliations of *Canthium* species placed under *Pyrostria* group B sensu Bridson (Vanguerieae, Rubiaceae) inferred from morphology and molecular data

**DOI:** 10.1186/s40529-014-0065-3

**Published:** 2014-09-11

**Authors:** Grecebio Jonathan D Alejandro, Carizza Marie M Magdaleno, Joseph Alvin T Pacia, Lyn D Paraguison, Kim Karlo C Quiogue, Annie Eliza D Wong, Krysten Marie R Yayen, Axel H Arriola

**Affiliations:** 1 College of Science, University of Santo Tomas, España, Manila 1015 Philippines; 2 Research Center for the Natural & Applied Sciences, University of Santo Tomas, España, Manila 1015 Philippines; 3 The Graduate School, University of Santo Tomas, España, Manila 1015 Philippines; 4 Department of Biological Sciences, College of Arts and Sciences, University of the East, 2219 C.M. Recto Ave, Manila, Philippines

**Keywords:** Bayesian analysis, ITS, Psydrax, Pyrostria, trnL-F, Vanguerieae

## Abstract

**Background:**

*Pyrostria sensu lato* (s.l.) is regarded as one of the polyphyletic group within Vanguerieae formerly comprising of *Pyrostria sensu stricto* (s.s.), *Pyrostria* group A and *Pyrostria* group B delineated by the number of locules and geographical occurrence. Recent molecular phylogenetic studies within the genus have narrowed its circumscription that resulted in the merging of *Pyrostria* group A and *Pyrostria* s.s. Although some species of *Pyrostria* group B were already transferred to *Pyrostria* s.s. and *Psydrax* based on morphology, other representatives of the group remain unsettled.

**Results:**

Bayesian and parsimony analysis of the combined ITS (nrDNA) and *trnL-F* (cpDNA) datasets showed a well-supported clade of the whole Vanguerieae containing four Philippine endemic representatives of *Pyrostria* group B. The placement of *Canthium oligophlebium, Canthium obovatifolium* and *Canthium ramosii* within *Pyrostria* s.s. (PP = 0.99; BS = 85%) is robustly supported likewise the affiliation of *C. gynochthodes* with *Psydrax* (PP = 0.94; BS = 85%). Morphological features shared by our species with *Pyrostria* s.s*.* and *Psydrax* further supports our molecular data.

**Conclusion:**

Our study supports the earlier hypothesis that *Canthium oligophlebium, C. obovatifolium* and *C. ramosii* should be placed under *Pyrostria* s.s. except for *C. gynochthodes* that grouped with *Psydrax*. Four new combinations are proposed in this study. The generic affiliations of other species of *Pyrostria* group B should be reinvestigated towards a more natural classfication in Vanguerieae.

**Electronic supplementary material:**

The online version of this article (doi:10.1186/s40529-014-0065-3) contains supplementary material, which is available to authorized users.

## Background

The tribe Vanguerieae has long been regarded as monophyletic since it is easily delineated from the rest of Rubiaceae by the presence of a unique type of pollen presenter above the style (Verdcourt [1987]; Bridson [1992]). Recent molecular phylogenetic studies of the tribe (Lantz et al. [2002]; Lantz and Bremer [2004], [2005]) support previous classifications of Bridson ([1985], [1986], [1992]) in reinstating *Keetia* E. Phillips and *Psydrax* Gaertn. and raising *Canthium* subg. *Afrocanthium* Bridson to a genus level. Meanwhile, *Vangueria* Juss. was recircumscribed and is distinguished from its close relatives by having inflorescences borne from where leaves have fallen, small bracteoles in secondary branch and large fruit with 2 to 5 locules. The genus *Canthium* was regarded as highly polyphyletic and redelimited to include species with supraaxillary spines.

Although major evolutionary lineages and new generic limits have been established within Vanguerieae utilizing morphology and molecular data, there are still understudied species. When Bridson ([1987]) reinstated the genus *Pyrostria* Comm. ex A. Juss., she lumped all *Pyrostria* species and representatives of *Canthium* with pair of persistent connate bracts (bracteate species) under *Pyrostria* s.l. and suggested informal groups (*Pyrostria* s.s., *Pyrostria* group A and *Pyrostria* group B) based on the number of locules and geographical occurrence. Both *Pyrostria* s.s. (pluri-locular ovary) and *Pyrostria* group A (bilocular ovary) have unisexual flowers and well represented in Madagascar, the latter extends from Africa to Arabia. Meanwhile, *Pyrostria* group B shares characters with *Pyrostria* group A in having unisexual flowers, bilocular ovaries, broad corolla tube, and 4-5 corolla lobes but the former radiates as far as SE Asia. The polyphyly of *Pyrostria* s.l*.* was initially addressed by Lantz and Bremer ([2004]) in recovering a clade composed mainly of dioecious species but failed to discussed further this group due to the poor internal support. Razafimandimbison et al. ([2009]) established a new generic delimitations using molecular data within the dioecious Vanguerieae by lumping species from *Pyrostria* group A to *Pyrostria* s.s. Their study, however, failed to include any species under *Pyrostria* group B and made an assumption that this group belongs to *Pyrostria* s.s. due to the presence of paired bracts which is a typical character for this genus. In an earlier study based on morphology, Utteridge and Davis ([2009]) transferred two SE Asian *Canthium* species belonging to *Pyrostria group* B (*C. brunnescens* Craib and *C. cochinchinense* Pierre ex Pit. in H.Lecomte) to *Pyrostria* s.s. Recently, Alejandro et al. ([2013]) transferred the Philippine endemic *C. subsessifolium* (Merr.) Merr. to *Pyrostria.* In contrast, *Plectronia amplifolia* Elmer informally placed under *Pyrostria* group B was transferred to *Psydrax* (Ruhsam et al. [2008]).

There are still left unresolved endemic Philippine species placed under *Pyrostria* group B probably associated with *Pyrostria* such as *C. brunneum* (Merr.) Merr., *C. ellipticum* (Merr.) Merr., *C. gynochthodes* Baill., *C. megacarpum* (Merr.) Merr., *C. obovatifolium* (Merr.) Merr., *C. oligophlebium* (Merr.) Merr., *C. ramosii* (Merr.) Merr., and *C. subcapitatum* (Merr.) Merr. Available herbarium materials of these species are scarce and lack reproductive parts for confirmation. In this study, four species of *Canthium* informally placed under *Pyrostria* group B: *C. gynochthodes, C. obovatifolium, C. oligophlebium*, and *C. ramosii* were collected and challenged their phylogenetic positions within Vanguerieae utilizing molecular sequence data. Furthermore, type specimens were meticulously examined to confirm our molecular results. The present study is a good contribution in understanding a more robust phylogenetic evolutionary trends and lineages within the tribe.

## Methods

### Taxon sampling

This study is based on the examination of herbarium sheets from various herbaria as well as field observation. *Canthium gynochthodes, C. obovatifolium, C. oligophlebium* and *C. ramosii* were collected based on their type protologues. Collected samples (herbarium specimens and preserved reproductive structures in 70% ethanol) were deposited at the USTH for accessioning. Leaf samples were dried in silica-gel for DNA extraction (Chase and Hills [1991]).

### Molecular methods

Genomic DNA was extracted from silica gel-dried leaf samples using the DNeasy Plant Minikit (Qiagen, Germany). The entire ITS region (including the 5.8S gene) was amplified and sequenced using the primer pair P17F/26-82R and P16F/P25R (Popp and Oxelman [2001]). Meanwhile, primer pair c/f were used for both amplification and sequencing of the *trnL-F* region (Taberlet et al. [1991]). DNA amplification was carried out following the work of Alejandro et al. ([2005], [2011]). Amplified DNA was purified using the QIA-quick Purification Kit (Qiagen, Germany). Purified DNA was sent to MACROGEN Inc., Seoul, South Korea for sequencing.

### Phylogenetic analysis

The ITS and *trnL-F* sequences were assembled and edited using the Codon Code Aligner version 3.0.1. Novel sequences of the four Philippine *Canthium* from each of the markers used were incorporated with several related sequences from the work of Lantz and Bremer ([2004]) taken from the GenBank (Table 1). *Ixora coccinea* L. and *Mussaenda erythrophylla* Schumach. & Thonn., considered as closely related to Vanguerieae were used as the outgroups. Sequences were aligned manually using Se-Al v.1.0al (Rambaut [1996]).

**Table 1:** **Nucleotide sequence database accession numbers of taxa used in this study**

Taxon	GenBank/EMBL Accession Number	
	ITS	***trnL-F***
*Afrocanthium burttii* (Bullock) Lantz	AJ617749	AJ620120
*Afrocanthium gilfillanii* (N.E. Br.) Lantz	AJ617751	AJ620123
*Afrocanthium keniense* (Bullock) Lantz	AJ617753	AJ620126
*Afrocanthium lactescens* (Hiern) Lantz	AJ617754	AJ620127
*Afrocanthium mundianum* (Cham. & Schltdl.) Lantz	AJ315107	AJ620128
*Afrocanthium parasiebenlistii* (Bridson) Lantz	AJ617756	AJ620130
*Afrocanthium pseudoverticillatum* (S. Moore) Lantz	AJ617758	AJ620132
*Afrocanthium siebenlistii* (K. Krause) Lantz	AJ617759	AJ620133
*Canthium ciliatum* (D. Dietr.) Kuntze	AJ617750	AJ620121
*Canthium coromandelicum* (Brum. f.) Alston	AJ315081	AJ620122
*Canthium glaucum* Hiern ssp. *glaucum*	AJ617752	AJ620124
*Canthium gynochthodes* ^1^	HG937666	HG937663
*Canthium inerme* (L.f.) Kuntze	AJ315120	AJ620125
*Canthium mrimaense* (Verdc.) Lantz	AJ617775	AJ620174
*Canthium obovatifolium* (Merr.) Merr.^2^	HG937664	HG937661
*Canthium oligocarpum* Hiern ssp. *Captum* (Bullock) Bridson	AJ617755	AJ620129
*Canthium oligophlebium* (Merr.) Merr.^3^	HG937665	HG937660
*Canthium ramosii* (Merr.) Merr.^4^	HG937667	HG937662
*Fadogia ancylantha* Schweinf.	AJ315103	AJ620136
*Fadogia arenicola* K.Schum. & K.Krause	AJ874981	AJ874943
*Fadogia tetraquetra* K. Schum. & K. Krause	AJ315099	AJ620139
*Fadogia triphylla* Baker	AJ874982	AJ874944
*Keetia gueinzii* (Sond.) Bridson	AJ315117	AJ620143
*Keetia lukei* Bridson	AJ617761	AJ620144
*Keetia venosa* (Oliv.) Bridson	AJ617762	AJ620145
*Keetia zanzibarica* (Klotzsch) Bridson ssp. *zanzibarica*	AJ315105	AJ620138
*Psydrax kraussioides* (Hiern) *Bridson*	AJ617786	AJ620157
*Psydrax livida* (Hiern) *Bridson*	AJ617769	AJ620158
*Psydrax locuples* (K. Schum.) *Bridson*	Aj617770	Aj620159
*Psydrax parviflora* (Afzel.) *Bridson*	Aj315110	AJ620162
*Pyrostria ampijoroense* (Arènes) Razafim., Lantz & B. Bremer	AJ617766	AJ719194
*Pyrostria hystrix* (Bremek.) Bridson	AJ315114	AJ620168
*Pyrostria major* (A. Rich. ex DC.) Cavaco	EU 584304	FN386344
*Pyrostria orbicularis* A. Rich. ex DC.	EU584285	FN386347
*Pyrostria phyllantoidea* (Baillon) Bridson	AJ315115	AJ620169
*Pyrostria revoluta* (Balf. f.) Razafim., Lantz & B. Bremer	AJ617776	AJ620176
*Pyrostria sarodranensis* Cavaco	EU584280	FN386366
*Pyrostria serpentina* Lantz, Klack. & Razafim.	EU584283	FN386350
*Vangueria infausta* Burchell	AJ617777	AJ620180
*Vangueria proschii* Briq.	AJ875009	AJ874975
*Vangueria parvifolia* Sond.	AJ315092	AJ620181
*Ixora coccinea* L.	AJ224826	AJ620117
*Mussaenda erythrophylla* Schumach. & Thonn.	AJ224823	AJ620116

Since vouchers of most taxa included in the study were published only the voucher information of the Philippine *Canthium* included in the study are provided as footnotes.

^1^Philippines, Province of Palawan, Arriola and Alejandro 12442 (USTH).

^2^Philippines, Province of Davao, Lemana and Alejandro BL10014 (USTH).

^3^Philippines, Province of Isabela, Lemana and Alejandro BA10017 (USTH).

^4^Philippines, Province of Quezon, Arriola and Alejandro S001 (USTH).

Bayesian inference (BI) was used to estimate phylogenetic positions of the Philippine endemic *Canthium* species. The analysis was carried out using the MrBayes v.3.1.2p software (Huelsenbeck and Ronquist [2001]; Ronquist and Huelsenbeck [2003]; Altekar et al. [2004]). Model selection for the best-performing evolutionary models were determined under three model selection criteria: a) Akaike Information Criterion (AIC) (Akaike [1974]), b) AICc (seconder order criterion of AIC, necessary for smaller samples) and c) the Bayesian Information Criterion (BIC) (Schwartz [1978]). The selected models were HKY and GTR + G for the ITS and *trnL-F*, respectively. In analyzing single marker, the best performing model was selected and one million generation was considered with a sample frequency of 1000 and four parallel chains. For combined analyses, model selection as well as the settings is similar with that of the single-marker analysis, however there were a total of three million running generations. Clades with posterior probability (PP) exceeding 0.95 were regarded as strongly supported.

Parsimony analysis was conducted using PAUP version4.0b (Swofford [2000]). Heuristic search was carried out to determine the most parsimonious trees utilizing a tree-bisection reconnection (TBR) branch swapping using 10,000 random addition sequences, with MULTREES option on. Consistency index (Kluge and Farris [1969]) and retention index (Farris [1989]) were calculated to determine if the data is far from being homoplasious. Bootstrapping was determined using 10,000 replicates, MULTREES option off, TBR branch swapping, and five random addition sequences. Clades receiving greater than 90% were considered strongly supported.

## Results

### Sequence characteristics

Table 2 shows the matrix characteristics of the separate and combined ITS and *trnL-F* data sets. The aligned matrix of the 43 taxa of the ITS region includes a total of 691 positions, 190 base pairs (bp) of which are phylogenetically informative. The 43 sequences of *trnL-F,* have a total of 1,002 positions, 43 bp of which are informative. The combined ITS/*trnL-F* of the 43 taxa with 1,693 characters generated a total of 233 bp informative characters.

**Table 2:** **Matrix characteristics of separate and combined datasets**

	ITS	***trnL-F***	Combined
Number of taxa	43	43	43
Number of included characters	691	1,002	1,693
Number of informative characters	190	43	233
Consistency index	0.56	0.93	0.64
Retention index	0.74	0.93	0.76

### Phylogenetic analysis

The tree topologies of the separate ITS (PP = 1.00; BS = 100%) and *trnL-F* (PP = 0.89; BS = 90%) analyses (trees not shown) revealed a monophyletic Vanguerieae. Both trees resolved the phylogenetic positions of *C. obovatifolium, C. oligophlebium* and *C. ramosii* in *Pyrostria* clade with high support in ITS (PP = 0.96; BS = 89%) and *trnL-F* (PP = 1.00; BS = 85%). However, both separate analyses failed to resolve the placement of *C. gynochthodes* within the tribe and polytomies were observed for members of *Canthium* s.s and *Psydrax*.

**Figure 1:**
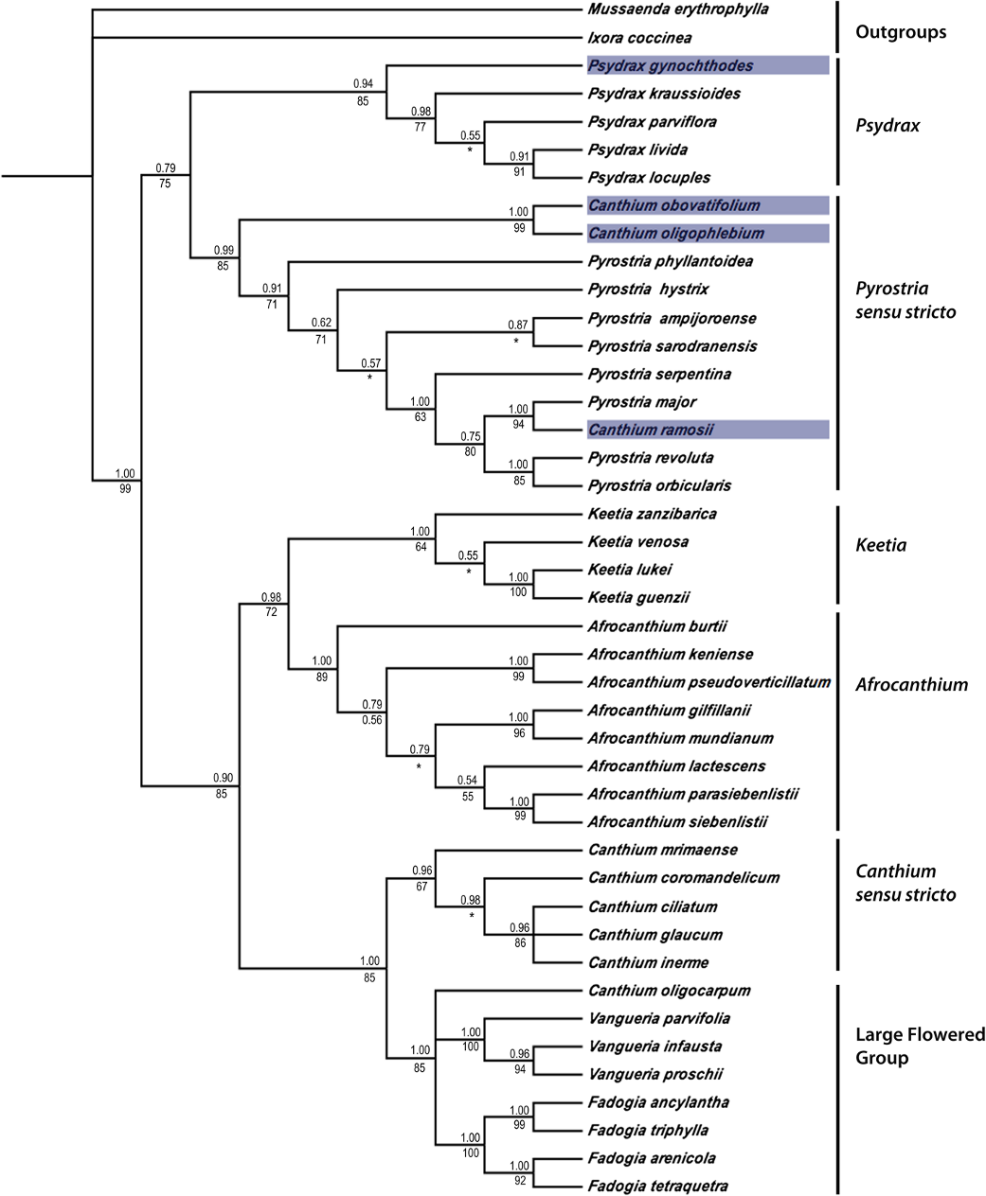
**Majority-rule consensus tree inferred from the Bayesian analysis of the combined ITS/** ***trnL-F*** **datasets of the 43 included taxa.** The results are congruent with the results of parsimony analysis except for the nodes marked with asterisks. Numbers above branches indicates Bayesian posterior probabilities and those below branches are parsimony bootstrap values. Species under study are highlighted in grey.

Bayesian and parsimony analyses of the combined ITS/*trnL-F* data sets (Figure 1) shows a robustly supported Vanguerieae (PP = 1.00; BS = 99%) (Figure 1). The majority rule consensus tree of the combined ITS/*trnL-F* data sets (Figure 1) supports the monophyly of the included genera and recovered tree topologies similar with Lantz and Bremer ([2004]). For instance, the monophyly of *Canthium* s.s. is supported (PP = 0.96; BS = 67%) and is closely related to the large flowered group (PP = 1.00; BS = 85%); *Keetia* (PP = 1.00; BS = 64%) and *Afrocanthium* (PP = 1.00; BS = 89%) as sister taxa is likewise supported (PP = 0.98; BS = 72%); and the monophyly of *Psydrax* (PP = 0.94; BS = 85%) and *Pyrostria* s.s. (PP = 0.99; BS = 85%) were also sustained. The combined data analysis agrees with single marker analyses on the close relatedness of *C. obovatifolium, C. oligophlebium* and *C. ramosii* with *Pyrostria* (PP = 0.99; BS = 85%). Meanwhile, *C. gynochthodes* is finally resolved within *Psydrax* (PP = 0.94; BS = 85%).

## Discussion

### Generic affiliations of species under Pyrostria group B

The results presented above clearly shows the polyphyly of *Canthium* as earlier observed by Lantz et al. ([2002]), Lantz and Bremer ([2004], [2005]) and Razafimandimbison et al. ([2009]). The four *Canthium* (*C. gynochthodes, C. obovatifolium, C. oligophlebium* and *C. ramosii*) should be excluded from *Canthium* s.s. since these species are spineless.

The phylogenetic position of *C. obovatifolium, C. oligophlebium* and *C. ramosii* within the *Pyrostria* clade was already anticipated due to the presence of a persistent, basally paired, connate to acuminate bracts as observed in our recent collections and available herbarium sheets. The synapomorphic characters of *Pyrostria* such as dioecious sexuality and fleshy corolla with trichomes in the throat (Lantz and Bremer [2004]) were also observed in our sampled *Canthium* species. The placement of these three *Canthium* species in *Pyrostria* s.l. was already suggested by Bridson ([1987]) but she was unsure of the placement in the genus due to their geographical occurrence falling outside the known range, i.e. at that time *Pyrostria* was considered to be a predominately Afro-Madagascan genus.

Although *Pyrostria* is mostly represented in Africa, Ruhsam et al. ([2008]) mentioned that the presence of SE Asian *Pyrostria* could probably be a disjunct part of their African relatives. There is a possibility that species under this genus may have undergone long range dispersal from Africa to Asia as in the case of *Mussaenda* L. (Alejandro et al. [2005]).

Meanwhile, the phylogenetic placement of *C. gynochthodes* within *Psydrax* does not support the earlier suggestion of Bridson ([1985]) in placing the species under *Pyrostria* s.l.. Bridson may have assigned this SE Asian species under *Pyrostria* group B due to the presence of bracts which resembles that of *Pyrostria* although Baillon ([1879]) did not mention the occurrence of this character. However, we examined herbarium sheets of *C. gynochthodes* [Gaerlan, F.J.M 0542753 (L, PNH); Romero, E.M. 0542751 (L, PNH); Soejarto, D.D. 0219674 (L, PNH); Arriola and Alejandro, 12442 (PNH, USTH); Arriola and Alejandro, 11057 (PNH, USTH)] and revealed that bracts exist in younger inflorescences but totally absent in older ones. The presence of bracts on young inflorescences of *C. gynochthodes* will not affect its close relatedness with *Psydrax.* According to Bridson ([1987]) bracts may be present in some representatives of *Psydrax*, however, it is distinctive from the paired connate bracts of *Pyrostria* which are rare in Vanguerieae. The presence of bracts is not a cardinal character to delimit *Psydrax*. For instance, Bridson ([1985]) mentioned of the occurrence of bracts in the Indian *Psydrax umbellata* (Whit.) Bridson and unnamed Malayan species. Furthermore, examination of *C. gynochthodes* revealed that it posseses other diagnostic features of *Psydrax* such as coriaceous leaf blades, keeled stipules with truncate to triangular stipular base and falcate stipular apex, reflexed anthers, long style always exceeding the corolla tube, longer than wide stigmatic knob, cartilaginous seed and a very shallow to nearly inconspicuous apical crest without a lid-like area in the pyrene (Bridson [1985]; Cheek and Sonke [2004]). Additionally, the occurrence of a unique insertion of 40 bp in the *trnL-F* region of *Psydrax* that is non-alignable with other species of Vanguerieae (Lantz and Bremer [2004]) exists in *C. gynochthodes*.

The close relatedness of species placed under *Pyrostria* group B with *Pyrostria* s.s. and *Psydrax* are supported by morphology and molecular data. Therefore, it is necessary to recollect the remaining species of *Pyrostria* group B to determine their correct generic affiliations within the tribe.

### Taxonomic treatment

**Figure 2:**
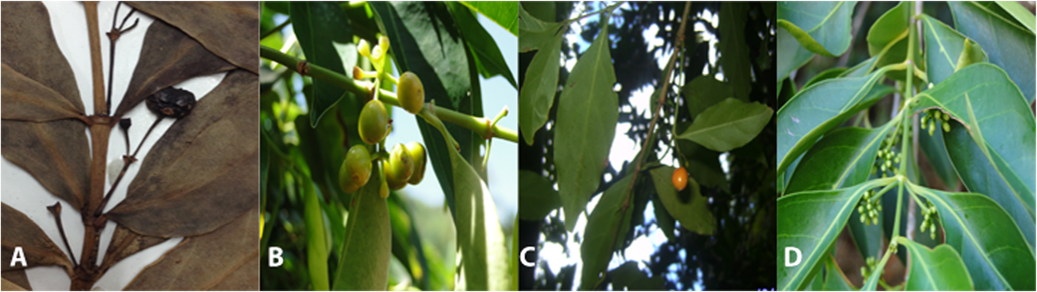
**Images of the four plant species included in the study.** **A.** *Pyrostria obovatifolia* fruiting branch; **B.** *Pyrostria oligophlebia* infructiscences; **C.** *Pyrostria ramosii* fruiting branch; **D.** *Psydrax gynochthodes* flowering branch.

We present here novel combinations of four species that were included in our study (Figure 2).

***Pyrostria obovatifolia*** (Merr.) Wong, Magdaleno & Alejandro, comb. nov. Basionym: *Canthium obovatifolium* (Merr.) Merr., Philipp. J. Sci. 35 (1928) 8. *Plectronia obovatifolia* Merr., Philipp. J. Sci. C12 (1917) 167. Philippines, Luzon, Tayabas Prov. Mount Dalindingan, Sept. 1916, *Ramos* and *Edano 26526* (holotype: PNH destroyed; lectotype: designated here K!; isolectotypes: US, HUH) (Figure 2A).

Shrub to small tree less than 3 m high; branches terete to a more or less quadrangular and glabrous. Leaves obovate, 3.5-7.5 × 1.0-3.0 cm, glabrous on both sides; apex rounded; base acute to acuminate; visible lateral nerves 3 to 4 on each side of the midrib; petiole 2.5-8.0 mm, glabrous. Stipules triangular to broadly ovate, 5.0-6.0 × 1.0 mm, glabrous on both sides. Female inflorescences axillary on 3.0-5.5 mm long peduncles, 6-flowered; peduncular bracts present, 3.0-5.5 mm long, triangular to broadly triangular, glabrous on both sides, enclosing the young inflorescence; pedicels erect, 3.0-4.0 mm long at flowering. Female flowers: calyx limb glabrous; tube 1.2- 2.5 mm long; lobes acuminate, 0.2 × 0.4 mm. Corolla 5-merous, white, glabrous outside; tube tubular, 0.8-1.2 mm long, hairs present at the throat; lobes broadly triangular, 2.0-2.5 × 1.0-1.2 mm, recurved. Stamens exerted, attached to corolla tube; anthers narrowly ovate to ovate, 0.3 mm long, exserted. Style including stigmatic knob 3.0-3.9 mm long; stigmatic knob 1 mm long, with a shallow cleft above, style not recessed into the stigmatic head; disk glabrous. Ovary 2-locular. Male flower unknown. Fruits ovoid 8.5-10.5 mm, glabrous.

**Distribution:-Luzon Island:** Ilocos Norte, Quezon

**Habitat:**-In secondary forest; 200-350 m altitude.

**Phenology:-** Flowering from March to June; Fruiting May to December

**Taxonomic notes:** This species approaches *P. subsessifolia* by its elliptic to ovate leaf shape but differs by its less conspicuous lateral nerves, shorter bracts and non- keeled fruits.

***Pyrostria oligophlebia*** (Merr.) Pacia, Quiogue & Alejandro, comb. nov. Basionym: *Canthium oligophlebium* (Merr.) Merr., Philipp. J. Sci. 35 (1928) 8. *Plectronia oligophlebia* Merr., Philipp. J. Sci. 17 (1921) 442. Philippines, Luzon, Rizal Prov. Mount Susong Dalaga, Aug. 1917, *Ramos* and *Edano 29342* (holotype, PNH destroyed; lectotype: designated here US; isolectotype: HUH, K !) (Figure 2B)

Shrub to small tree, less than 5.0 m high; branches quadrangular to more or less terete and glabrous. Leaves broadly lanceolate to oblong, 3.0-5.5 × 1.0-2.0 cm, glabrous on both sides; apex acute; base acute; visible lateral nerves 2 to 4 on each side of the midrib; petiole 0.7-1.0 mm, glabrous. Stipules broadly triangular to ovate, 1.0-2.0 × 1.0 mm, glabrous on both sides. Female inflorescences axillary on a glabrous peduncle less than 2 mm long, 7-12 flowered; peduncular bracts present, 2.5-3.0 mm long, triangular to broadly triangular, glabrous on both sides, enclosing the young inflorescence; pedicels erect, 4.0-5.0 mm long at flowering, persistent. Female flowers: calyx limb glabrous 2-2.7 mm long; lobes shortly toothed, 0.1 × 0.3 mm. Corolla 4-merous, white, glabrous outside; tube tubular, 0.8-1.2 mm long, hairs present at the throat; lobes broadly ovate, 2.0-2.5 × 1.0-1.2 mm. Stamens attached to corolla tube adjacent to the throat; anthers ovate, 0.3 mm long, exserted. Style including stigmatic knob 1.0-2.5 mm long; stigmatic knob 1 mm long, with a shallow cleft above; disk glabrous. Ovary 2-locular. Male flower unknown. Fruits ovoid 6.0-6.5 mm, glabrous with distinct indentation when dry.

**Distribution:-Luzon Island:** Rizal; **Mindanao Island:** Davao

**Habitat:**-In secondary forest; 500-900 m altitude.

**Phenology:-** Flowering from March to December; Fruiting from Septmber to February

**Taxonomic notes**: The smaller and fewer nerved leaves of *P. oligophlebia* approaches *P. gynochthodes*. However, *P. oligophlebia* differs from the latter by having persistent pair of bracts, many-flowered inflorescences and a longer petioles, peduncles and pedicel.

***Pyrostria ramosii*** (Merr.) Arriola, Paraguison & Alejandro, comb. nov.

Basionym: *Canthium ramosii* (Merr.) Merr., Philipp. J. Sci. 35 (1928) 9. *Plectronia ramosii* Merr., Philipp. J. Sci. 7 (1921) 443. Philippines, Luzon, Tayabas Prov., Mount Umiray, June 1917, *Ramos* and *Edano 28973* (holotype, PNH destroyed; lectotype: designated here NY!; isolectotype: K!) (Figure 2C)

Tree less than 9 m high; branches terete, glabrous. Leaves broadly lanceolate to oblong, 2.5-9.5 × 1.5-3.9 cm, glabrous on both sides; apex triangular to acuminate; base attenuate; visible lateral nerves 3 to 4 on each side of the midrib; petiole 1.5-2.0 mm, glabrous. Stipules triangular, 3.5-4.0 × 1.5-2.0 mm, glabrous on both sides. Inflorescences 4 flowered umbels; pedicels erect, 6.5-7.0 mm, persistent glabrous, peduncular bracts present, 0.5-1.5 mm long, triangular, glabrous on both sides. Infructiscence glabrous; stalk glabrous. Fruits ovoid to didymous 6.5-8.5 mm, 2 -celled, glabrous.

**Distribution:-Luzon Island:** Quezon

**Habitat:**-In secondary forest; 200-350 m altitude.

**Phenology:-** Fruiting August to December

**Taxonomic notes:** *Pyrostria oligophlebia* and *P. ramosii* closely resemble each other due to their oblong shape leaf with acuminate apex. However, the latter have longer peduncles and few (2-4) flowered umbellate inflorescences as compared to the numerous (7-12) flowered inflorescence of the former.

***Psydrax gynochthodes*** (Baill.) Arriola, Yayen & Alejandro, comb. nov.

Basionym: *Plectronia gynochthodes* (Baill.) Merr., Enum. Philipp. Fl. Pl. 3 (1923) 536. *Canthium gynochthodes* Baill., Adansonia 12 (1879) 199. Philippines, Luzon, Batangas Prov., 1917, *Cuming 1848* (holotype, K!) (Figure 2D)

Tree, less than 7 m tall; branches flattened to subterete, glabrous. Stipules glabrous on both sides, triangular to ovate, 2-4.5 mm, keel prominent on the abaxial side. Petioles 1.0-5.0 × 0.3-0.5 mm, glabrous; leaf blades leathery, elliptic to elliptic-oblong or obovate, 3-8.5 × 1.5-5 cm, base acute to broadly acute, apex obtuse, glabrous throughout, glossy; lateral nerves 3-4 on each side of the midrib, domatia present. Inflorescences axillary, 10-12(-several) flowered, glabrous; peduncle 0.8-2.5 mm, glabrous; pedicel 4.0-10.0 mm, puberulent. Calyx tube infundibuliform, 2-3 × 2.0-2.5 mm, glabrous; lobes triangular 1.5-2.0 mm. Corolla tube infundibuliform, 1.0-1.5 mm, glabrous outside, with ring of white hairs inside; lobes 4, triangular, glabrous outside and inside. Anthers 4, reflexed. Style 2-6 mm, exceeding corolla tube, stigmatic-knob longer than wide, 0.3 × 0.2 mm, bifid. Ovary bilocular, 1-ovule per locule. Fruits ovoid to didymous, distinctly broader than long, 7.5-8.0 × 9-10 mm, green when young, glabrous, calyx limb persistent; Seeds 2, obliquely ovoid, ventrally flattened, 2-5.5 × 3 mm, apical crest very shallow to nearly inconspicuous, pyrene cartilaginous.

**Distribution:-** Philippines, Taiwan

**Habitat:**-In secondary forest; 500-900 m altitude.

**Phenology:-** Flowering from March to June; Fruiting from June to December

**Taxonomic notes:** *Psydrax gynochthodes* is comparable with *P. obovatifolia, P. oligophlebia* and *P. ramosii*. However it is delineated from the three species due to the absence of persistent acuminate-connate bracts. For the above reason, we do not agree with the observation of Bridson ([1987]) that *P. gyncochthodes* is closely associated with *P. villarii* Vidal and that the two species should be synonymize. Moreover, *P. gynochthodes* can be distinguised from the Philippine *P. amplifolia* by its smaller, thicker and darker glossy green leaves, umbellate to cymose inflorescences, longer peduncles and smaller fruits.

## Conclusion

The generic affiliations of four species previously hypothesized under *Pyrostria* group B have been resolved based on morphology and molecular sequence data. We formally proposed three novel combinations in *Pyrostria* and a *Psydrax*. Other species of *Pyrostria* group B should be reinvestigated towards a more natural classfication in Vanguerieae. Furthermore, large number of *Pyrostria* and bracteate species temporarily placed under *Canthium* s.l should be sampled to fully understand the evolutionary dispersal of the genus.

## Authors’ contributions

GJDA and AHA drafted the manuscript; All authors participated in sample collection, molecular work and taxonomic treatment. All authors read and approved the final manuscript.

## Authors’ original submitted files for images

Below are the links to the authors’ original submitted files for images. Authors’ original file for figure 1 Authors’ original file for figure 2

## References

[CR1] Akaike H (1974). A new look at the statistical model identification. IEEE Trans Autom Control.

[CR2] Alejandro GJD, Razafimandimbison SG, Liede-Schumann S (2005). Polyphyly of *Mussaenda* inferred from ITS and *trnT-F* data and its implication for generic limits in Mussaendeae (Rubiaceae). Am J Bot.

[CR3] Alejandro GJD, Meve U, Mouly A, Thiv M, Liede-Schumann S (2011). Molecular phylogeny and taxonomic revision of the Philippine endemic *Villaria* Rolfe (Rubiaceae). Plant Syst Evol.

[CR4] Alejandro GJD, Arenas EH, Cremen CM, Arriola AH (2013). A new record of *Pyrostria* (Vanguerieae-Rubiaceae) from the Philippines inferred from molecular and morphological data. Phil J Syst Bio.

[CR5] Alterkar G, Dwarkadas S, Huelsenbeck JP, Ronquist F (2004). Parallel metropolis coupled Markov chain Monte Carlo for Bayesian phylogenetic inference. Bioinformatics.

[CR6] Baillon HE (1879) *Canthium gynochthodes*. Adansonia 12:199 Baillon HE (1879) Canthium gynochthodes. Adansonia 12:199

[CR7] Bridson DM (1985). The reinstatement of *Psydrax* (Rubiaceae, subfam. Cinchonoideae tribe Vanguerieae) and a revision of the African species. Kew Bull.

[CR8] Bridson DM (1986). The reinstatement of the African genus *Keetia* (Rubiaceae subfam. Cinchonoideae, tribe Vanguerieae). Kew Bull.

[CR9] Bridson DM (1987). Studies in African Rubiaceae- Vanguerieae: a new circumscription of *Pyrostria* and a new subgenus, *Canthium* subgen. *Bullockia*. Kew Bull.

[CR10] Bridson DM (1992). The genus *Canthium* (Rubiaceae- Vanguerieae) in tropical Africa. Kew Bull.

[CR11] Chase MW, Hills HH (1991). Silica gel: an ideal material for preservation of leaf samples for DNA studies. Taxon.

[CR12] Cheek M, Sonke B (2004). *Psydrax bridsoniana* (Rubiaceae), a new species of tree from western Cameroon. Kew Bulletin.

[CR13] Farris JS (1989). The retention index and the rescaled consistency index. Cladistics.

[CR14] Huelsenbeck JP, Ronquist F (2001). MRBAYES: Bayesian inference of phylogenetic trees. Bioinformatics.

[CR15] Kluge AG, Farris JS (1969). Quantitative phyletics and the evolution of anurans. Syst Zool.

[CR16] Lantz H, Bremer B (2004). Phylogeny inferred from morphology and DNA data: characterizing well-supported groups in Vanguerieae (Rubiaceae). Bot J Linn Soc.

[CR17] Lantz H, Bremer B (2005). Phylogeny of the complex Vanguerieae (Rubiaceae) genera *Fadogia*, *Rytigynia*, and *Vangueria* with close relatives and a new circumsumption of Vangueria. Plant Syst Evol.

[CR18] Lantz H, Andreasen K, Bremer B (2002). Nuclear rDNA ITS used to construct the first phylogeny of Vanguerieae (Rubiaceae). Plant Syst Evol.

[CR19] Popp M, Oxelman B (2001). Inferring the history of the polyploid *Silene aegaea* (Caryophyllaceae) using plasmid and homoeologous nuclear DNA sequences. Mol Phylogenet Evol.

[CR20] Rambaut A (1996) Se-Al v1.0a1. . Accessed 15 May 2011, [http://tree.bio.ed.ac.uk/software/seal/] Rambaut A (1996) Se-Al v1.0a1. . Accessed 15 May 2011

[CR21] Razafimandimbison SG, Lantz H, Mouly A, Bremer B (2009). Evolutionary trends, major lineages and new generic limits in the dioecious group of the tribe Vanguerieae (Rubiaceae): insights into the evolution of functional dioecy. Ann Mo Bot Gard.

[CR22] Ronquist F, Huelsenbeck JP (2003). MrBayes 3: Bayesian phylogenetic inference under mixed models. Bioinformatics.

[CR23] Ruhsam M, Govaerts R, Davis AP (2008). Nomenclatural changes in preparation for a World Rubiaceae Checklist. Bot J Linn Soc.

[CR24] Schwartz G (1978). Estimating the dimensions of a model. Ann Stat.

[CR25] Swofford DL (2000). PAUP*: Phylogenetic Analysis Using Parsimony (*and other methods) version 40b.

[CR26] Taberlet P, Gielly L, Pautou G, Bouvet J (1991). Universal primers for amplication of three non-coding regions of chloroplast DNA. Pl Mol Biol.

[CR27] Utteridge TMA, Davis AP (2009). Two new combinations in *Pyrostria* (Rubiaceae-Vanguerieae) from Thailand. Kew Bull.

[CR28] Verdcourt B (1987). Notes on African Rubiaceae: Vanguerieae. Kew Bull.

