# Supplementary figures and images for: Generic affiliations of Canthium species placed under Pyrostria group B sensu Bridson (Vanguerieae, Rubiaceae) inferred from morphology and molecular data
Source: Bot Stud. 2014 Sep 11;55:65. doi: 10.1186/s40529-014-0065-3 (PMC5432757; doi:10.1186/s40529-014-0065-3)

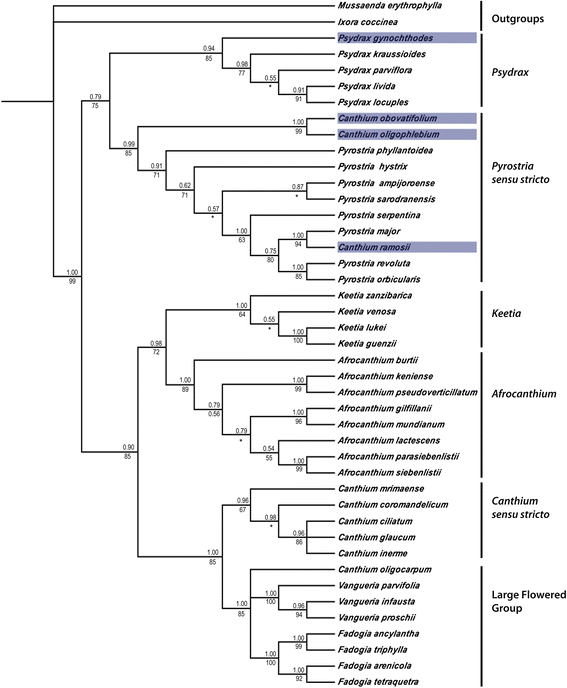

Supplement: Supplementary file 1 — Authors’ original file for figure 1 [file 40529_2014_9065_MOESM1_ESM.gif]

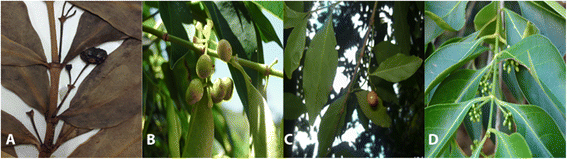

Supplement: Supplementary file 2 — Authors’ original file for figure 2 [file 40529_2014_9065_MOESM2_ESM.gif]
